# Differences in clinicopathological characteristics, treatment, and survival outcomes between older and younger breast cancer patients

**DOI:** 10.1038/s41598-021-93676-w

**Published:** 2021-07-12

**Authors:** Hikmat Abdel-Razeq, Sereen Iweir, Rashid Abdel-Razeq, Fadwa Abdel Rahman, Hanan Almasri, Rayan Bater, Ayat Taqash, Hadeel Abdelkhaleq

**Affiliations:** 1 Department of Internal Medicine, King Hussein Cancer Center, School of Medicine, University of Jordan, Queen Rania Al Abdullah Street, P.O. Box: 1269, Amman, 11941 Jordan; 2 School of Medicine, University of Jordan, Amman, Jordan; 3 Office of Scientific Affairs and Research, King Hussein Cancer Center, Amman, Jordan; 4 Istishari Hospital, Amman, Jordan; 5 Department of Radiation Oncology, King Hussein Cancer Center, Amman, Jordan

**Keywords:** Cancer, Oncology

## Abstract

In developing countries, breast cancer is diagnosed at a much younger age. In this study we investigate the dichotomies between older and young breast cancer patients in our region. The study involved two cohorts; older patients (≥ 65 years, n = 553) and younger ones (≤ 40 years, n = 417). Statistical models were used to investigate the associations between age groups, clinical characteristics and treatment outcomes. Compared to younger patients, older patients were more likely to present with advanced-stage disease (20.6% vs. 15.1%, *p* = .028). However, among those with non-metastatic disease, younger patients tended to have more aggressive pathological features, including positive axillary lymph nodes (73.2% vs. 55.6%, *p* < .001), T-3/4 (28.2% vs. 13.8%, *p* < .001) and HER2-positive disease (29.3% vs. 16.3%, *p* < .001). The 5-year overall survival (OS) rate was significantly better for the younger (72.1%) compared to the older (67.6%), *p* = .035. However, no significant difference was observed in disease-free survival (DFS) between the two groups.In conclusion, younger patients with breast cancer present with worse clinical and pathological features, albeit a better OS rate. The difference in DFS between the two groups was not insignificant, suggesting that older women were more likely to die from non-cancer related causes.

## Introduction

In both developing and developed countries, breast cancer (BC) is the most common cancer in women and is the second most commonly diagnosed type of cancer overall. Worldwide, more than 2.0 million new cases are diagnosed annually, accounting for almost 25% of all new cancers in women^1,2^. One of the frequently investigated risk and prognostic factors in BC is the possible relationship a patient’s age has with the tumor’s features and treatment outcomes. Epidemiological data had indicated that, despite the increased risk of BC diagnosis with age^3^, patients younger than 40 years-old demonstrate more aggressive disease and thus are at a higher risk of recurrence and disease-related mortality as well^4–7^. Contrarily, older patients tend to present with multiple comorbidities that complicate their outcomes and influence their treatment options and decisions^8,9^.

The situation in Jordan, and that of neighboring countries in the region, is a unique one in that patients are diagnosed with breast cancer at younger age compared to patients in Western societies. According to the latest report from Surveillance, Epidemiology, and End Results Medicare database (SEER), the median age of diagnosis for BC patients in the United States is 62 years^10^, while the mean age of diagnosis for Jordanian patients is 51–52 years old, as reported by the Jordanian national cancer registry^11^. A cross-sectional study conducted by King Hussein Cancer Center in Jordan in 2019, which is the primary cancer treatment center of the country, characterized the clinical features of all BC patients older than or equal to 65 years and revealed that the older patients had a 67.6% overall survival rate, only nodal metastasis was significantly associated with their survival, and they were not treated aggressively, with less than a third receiving chemotherapy^12^. On the other hand, a similar study that was performed at our institution on younger patients, aged ≤ 40 years, revealed that poor pathological characteristics such as lymph node involvement and lymphovascular invasion were prevalent in the population and were significantly associated with the overall and disease-free survival^13^.

For this study, we compared the clinical characteristics and outcomes of the older group of postmenopausal BC patients to the clinicopathological features and outcomes of the younger cohort using the data that was compiled for the two aforementioned cohorts^12,13^. This study will provide a valuable update on the use of age as a prognostic factor for BC patients, particularly given the large age gap between the two cohorts, as well as novel insight on a population develops the disease at an untypically younger age.

## Methods

### Study population

The clinical and demographical characteristics and outcomes of pathologically confirmed BC patients aged 40 years and younger between 2006 and 2013 were extracted from the hospital databases and medical records at our institute for a previous study conducted by the authors in 2019^13^. Likewise, a study published earlier this year entailed the collection of clinicopathological characteristics and treatment outcomes of BC patients aged 65 years and older, who had been diagnosed with pathologically confirmed BC between 2006 and 2016^12^. A master-database of the information obtained for the two studies was created; wherein coded cases of 417 adult patients who were 40 years old or younger at the time of diagnosis were labeled as belonging to the ‘young’ cohort, while 553 cases of patients who were 65 years or older at the time of diagnosis were designated as the ‘older’ cohort. Their survival status was identified at the start of the study. The patients were treated in accordance with institutional clinical practice guidelines and international standards. Treatment plans were discussed and approved at weekly multidisciplinary meetings at our institutions. The King Hussein Cancer Center Institutional Review Board (IRB) reviewed and approved the study and due to the retrospective nature of the study, patients’ consent was waived by the same committee.

### Statistical analysis

The Chi-square and Fischer’s exact testing methods were used to compare the frequencies of the clinical characteristics and surgical management plans of patients of the younger cohort to those of the older patients, while the non-parametric test was used to calculate the statistical difference of tumor size between the two cohorts. Last follow up was in January 2019 and survival curves were created using the Kaplan–Meier method to estimate the overall survival (OS), defined as the time from date of diagnosis to death from any cause or last follow up, and the disease-free survival (DFS), defined as the time from date of diagnosis to date of first local, regional or distal recurrence, or death by any cause without evidence of disease. The log-rank test and weighted log rank test were implemented to identify the statistical difference in the mean survival of the young and older BC patients. Multivariate analysis was done for the significant factors using Cox proportional hazards regression model. A *p* value of ≤ 0.05 was considered as statistically significant in all analyses. All statistical analyses were conducted using SAS version 9.4 (SAS Institute Inc., Cary, NC).

#### Ethics declarations

This research was conducted retrospectively from data obtained for clinical purposes and was carried in accordance with the 1964 Helsinki Declaration and its later amendments and comparable ethical standards. The IRB of King Hussein Cancer Center approved the study and waived the requirement of informed consent.

## Results

### Clinicopathological characteristics

**Figure 1:**
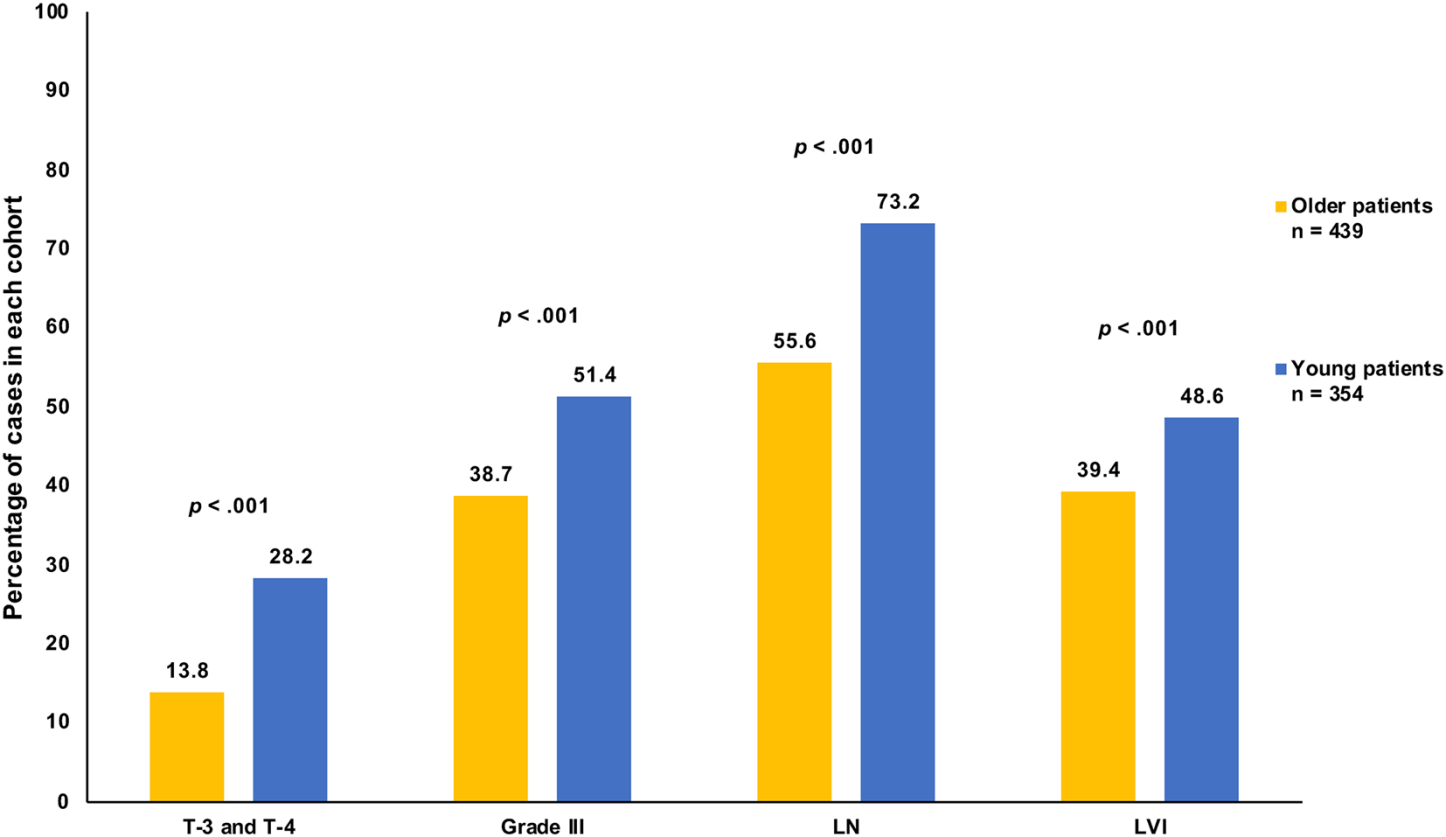
Clinical characteristics of M0 older and young breast cancer patients. LN = lymph node status, LVI = lymphovascular invasion.

During the study period, a total of 970 patients were included in the final analysis. Compared to younger patients (n = 417), older patients (n = 553) were more frequently diagnosed with distal metastasis (M1); 20.6% compared to 15.1%, *p* = 0.028. Among patients with non-metastatic disease (M0), younger patients were more likely to present with nodal metastasis (73.2% versus 55.6%, *p* < 0.001), grade-III tumors (51.4% versus 38.7%, *p* < 0.001), lymphovascular invasion (48.6% vs 39.4%, *p* < 0.001), and more likely to have T-3 (22.3%) and T-4 (5.9%) disease than were the older patients at 10.9% and 2.9%, respectively, *p* < 0.001 (Fig. 1). However, older patients were more likely to be diagnosed with invasive lobular carcinoma (ILC) at 10.8%, compared to only 4.8% of the young, *p* < 0.001. Further details of both clinical and pathological variables are summarized in Table 1.

**Table 1:** Patients' characteristics.

Clinical factor n (%)		Total (N = 970)	Older patients (n = 553)	Young patients (n = 417)	*p*
Tumor histology	IDC	862	466 (84.3%)	396 (95.0%)	*p* < .001
	ILC	80	60 (10.8%)	20 (4.8%)	
	Others^a^	28	27 (4.9%)	1 (0.2%)	
M stage	M0	793	439 (79.4%)	354 (84.9%)	.028
	MI	177	114 (20.6%)	63 (15.1%)	
Triple negative	No	883	506 (91.5%)	377 (90.4%)	.114
	Yes	76	38 (6.9%)	38 (9.1%)	
	NA	11	9 (1.6%)	2 (0.5%)	
Triple positive	No	804	464 (83.9%)	340 (81.5%)	*p* < .001
	Yes	105	44 (8.0%)	61 (14.6%)	
	NA	61	45 (8.1%)	16 (3.8%)	
HER2	Negative	679	411 (74.3%)	268 (64.3%)	*p* < .001
	Positive	212	90 (16.3%)	122 (29.3%)	
	NA	79	52 (9.4%)	27 (6.5%)	
ER	Negative	187	87 (15.7%)	100 (24.0%)	*p* < .001
	Positive	774	458 (82.8%)	316 (75.8%)	
	NA	9	8 (1.4%)	1 (0.2%)	
PR	Negative	217	105 (19.0%)	112 (26.9%)	.002
	Positive	744	440 (79.6%)	304 (72.9%)	
	NA	9	8 (1.4%)	1 (0.2%)	
ER or PR positive	No	162	81 (14.6%)	81(19.4%)	.048
	Yes	808	472 (85.4%)	336 (80.6%)	
ER and PR negative	No	817	480 (86.8%)	337 (80.8%)	.011
	Yes	153	73 (13.2%)	80 (19.2%)	

Clinical factor n (%)		Total (n = 793)	Older patients (n = 439)	Young patients (n = 354)	*p*
**For M0 cases only**					
T stage	T-1	206	114 (25.9%)	92 (26.0%)	*p* < .001
	T-2	391	230 (52.4%)	161 (45.5%)	
	T-3	127	48 (10.9%)	79 (22.3%)	
	T-4	34	13 (2.9%)	21 (5.9%)	
	Tis	2	2 (0.5%)	0	
	Tx	18	17 (3.9%)	1 (0.3%)	
	NA	15	15 (3.4%)	0	
Grade	I	47	38 (8.7%)	9 (2.5%)	*p* < .001
	II	389	226 (51.5%)	163 (46.0%)	
	III	352	170 (38.7%)	182 (51.4%)	
	NA	5	5 (1.1%)	0	
Lymphovascular invasion	Negative	384	204 (46.5%)	180 (50.8%)	*p* < .001
	Positive	345	173 (39.4%)	172 (48.6%)	
	NA	64	62 (14.1%)	2 (0.6%)	
Nodal metastasis	Negative	273	178 (40.5%)	95 (26.8%)	*p* < .001
	Positive	503	244 (55.6%)	259 (73.2%)	
	NA	17	17 (3.9%)	0	
Tumor size in cm	Mean (95% CI)		3.0 (2.9–3.2)	3.2 (3.0–3.4)	.6768
	Median (Range)		2.5 (0.0–13.0)	2.8 (0.0–13.0)	

IDC = invasive ductal carcinoma; ILC = invasive lobular carcinoma; NA = not available; M = Metastasis; ER = Estrogen receptors; PR = Progesterone receptors; HER2 = Human epidermal growth factor receptor 2; T = Tumor size, ER = Estrogen receptors; PR = Progesterone receptors.

^a^‘Others’ refers to instances when patient tumor histology was recorded as neuroendocrine carcinoma or inflammatory mammary cancer.

The hormone receptor status of patients in both cohorts differed significantly as well. Compared to younger patients, tumors in the older ones were more likely to be estrogen receptor (ER)-positive (82.8% versus 75.8%, *p* = 0.001) and progesterone receptor (PR)-positive (79.6% versus 72.9%, *p* = 0.002). Additionally, younger patients had a significantly higher percentage of human epidermal growth factor receptor 2 (HER2)-positive disease (29.3%) compared to 16.3% of older patients, *p* < 0.001. Moreover, younger patients had a significantly higher rate (14.6%) of triple-positive BC (positive ER, PR, and HER2), than did the older patients at 8.0%, *p* < 0.001. However, there was no statistically significant difference between the rates of triple-negative disease in the two cohorts; 6.9% of the older patients and 9.1% of the young patients, *p* = 0.114 (Table 1).

### Differences in surgical treatment

**Figure 2:**
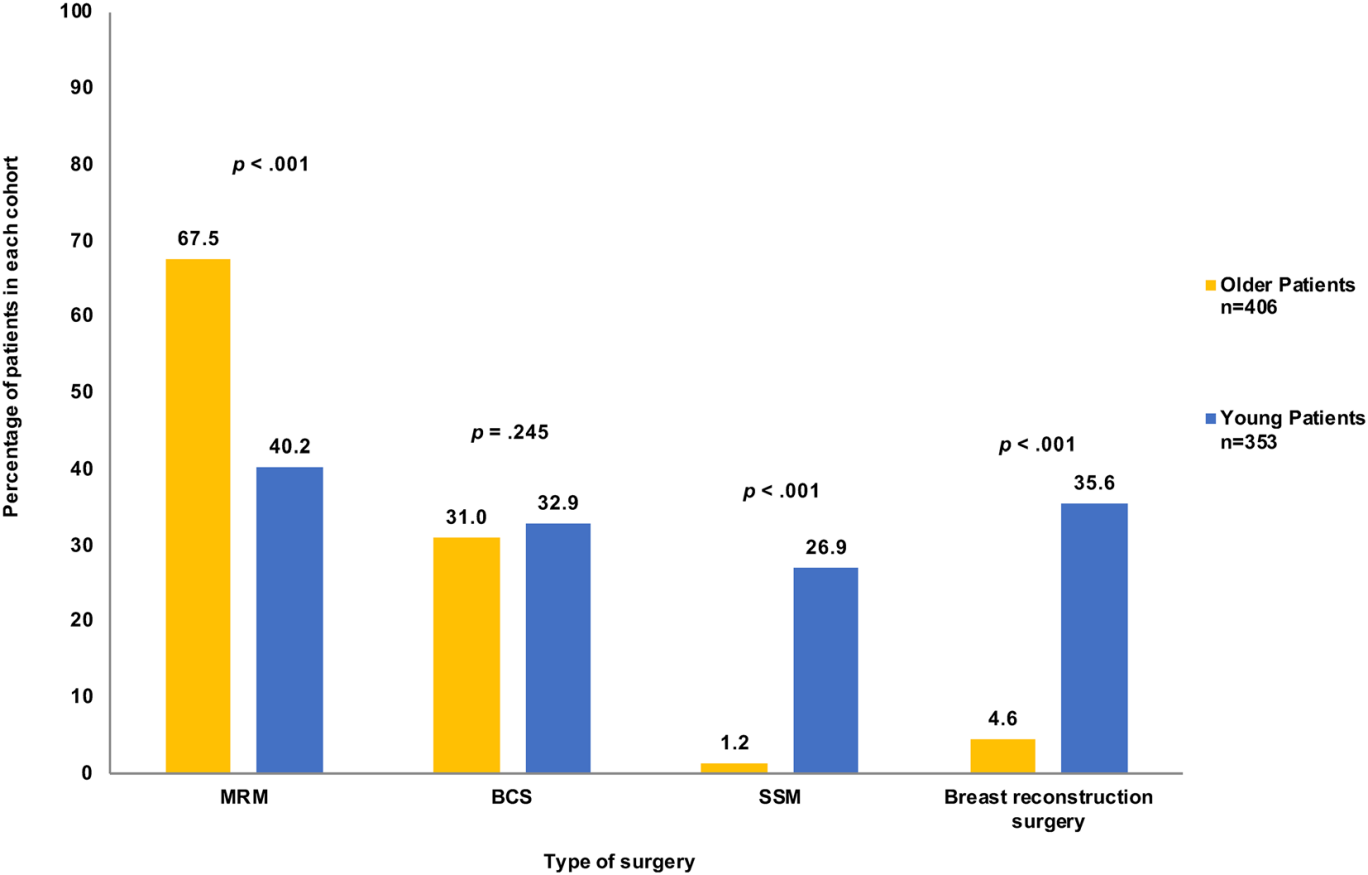
Difference in surgical management in patients with nonmetastatic (M0) disease. MRM = modified radical mastectomy, BCS = breast conserving surgery SSM = skin-sparing and/or nipple-sparing mastectomy.

A higher percentage of patients from the young cohort underwent any type of surgical treatment (85.4%) when compared to the older group (74.9%), *p* < 0.001. The differences in choice of surgery varied according to each type of surgery; for instance, a larger fraction of older M0 patients opted for modified radical mastectomy (MRM) (67.5%) than did younger patients (40.2%) (*p* < 0.001), while breast conserving surgery (BCS) was performed on almost a third in each cohort, *p* = 0.245. Skin-sparing, with or without, nipple-sparing mastectomies (SSMs) were more frequently carried out on younger patients (26.9%) than they were on the older patients (1.2%), *p* < 0.001. Moreover, there was a highly significant difference in the frequency of breast reconstruction surgery between the two cohorts; 35.6% of the younger nonmetastatic patients had the surgery, while only 4.9% of the older group of patients did, *p* < 0.001 (Fig. 2).

### Survival outcomes

**Figure 3:**
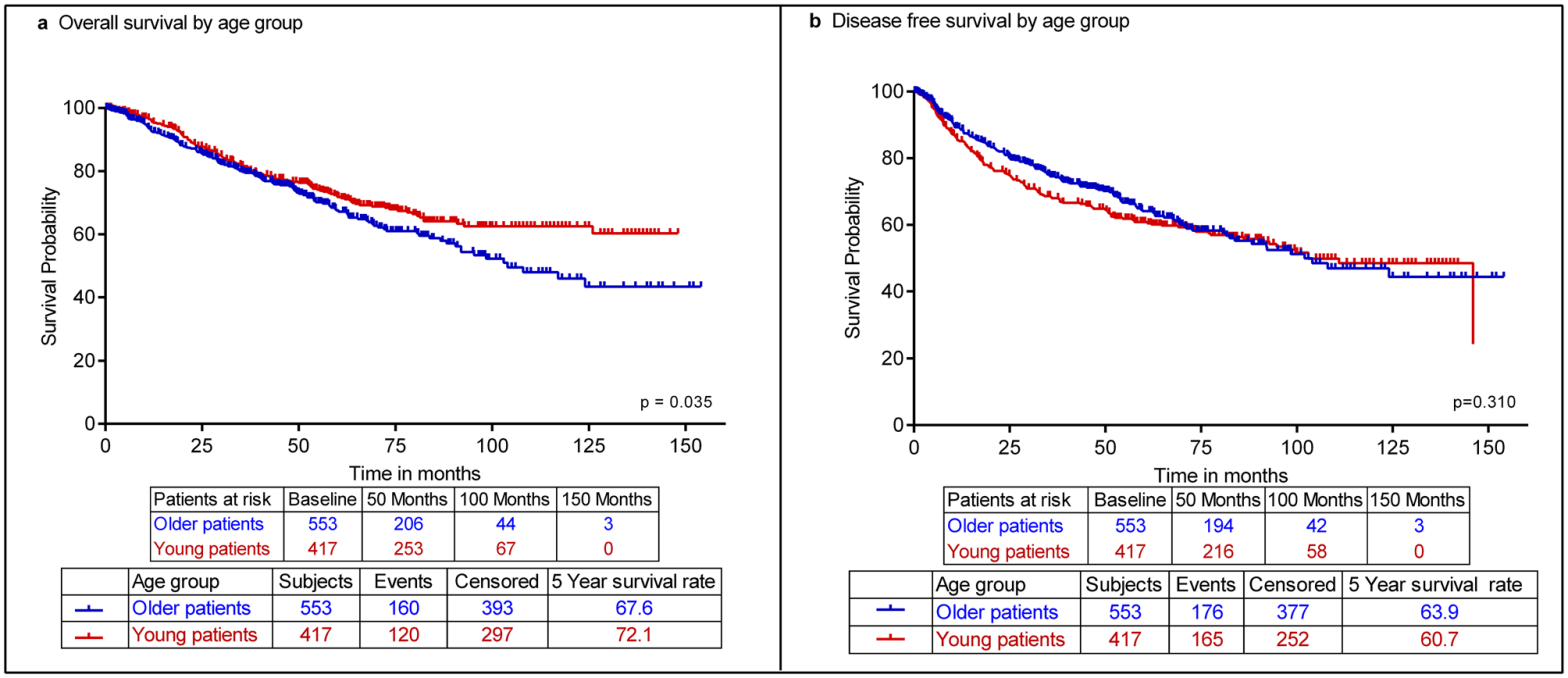
Overall and disease-free survival of older and young breast cancer patients. (**a**) Kaplan–Meier plot showing significant difference in overall survival by age group, log-rank *p* = .035. (**b**) Kaplan–Meier plot showing none-significant difference in disease-free survival by age group, log-rank *p* = .310.

**Figure 4:**
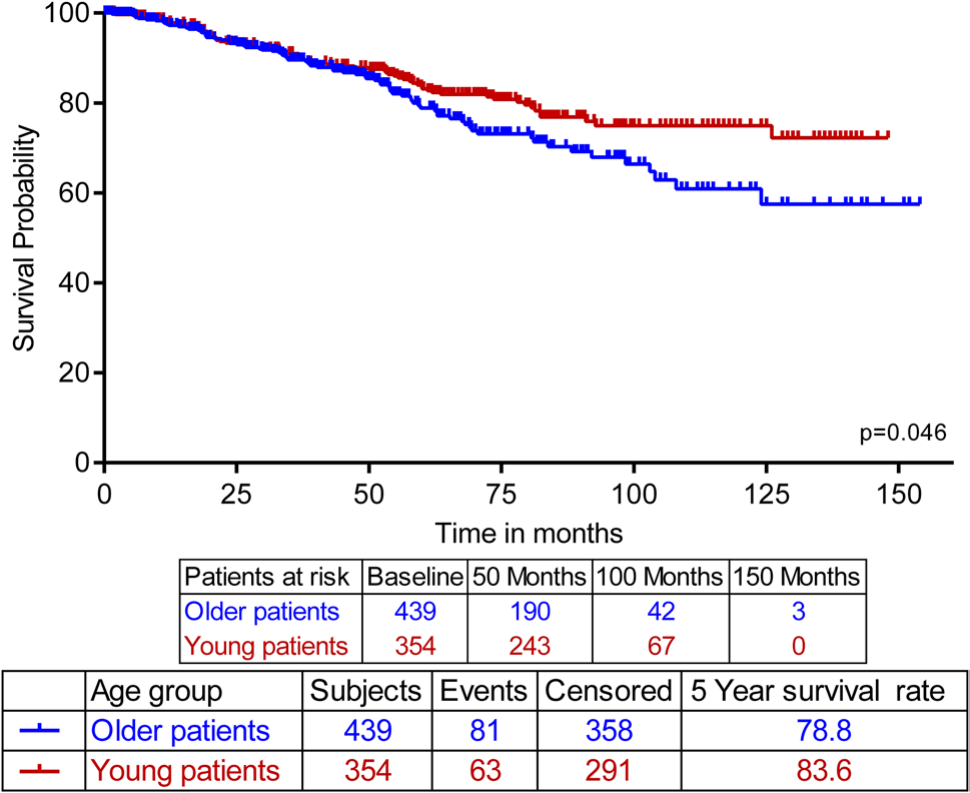
Overall survival of M0 breast cancer patients according to age group. Kaplan–Meier plot shows that the M0 older patients had a significantly worse overall survival rate with a log-rank *p* = .046.

After a median follow-up time of 59 months for the younger patients and 45 months for the older ones, the 5-year OS rate of the older patients was 67.6% compared to 72.1% for the younger patients, *p* = 0.035 (Fig. 3a). However, the 5-year DFS rates of the two cohorts were not statistically different at 63.9% for the older group and 60.7% for the young patients, *p* = 0.31 (Fig. 3b). We also studied the difference in survival of both groups of patients with non-metastatic disease; the 5-year OS among the younger patients was significantly higher at 83.6% compared to 78.8% among the older patients, *p* = 0.046 (Fig. 4).

Additionally, the multivariate Cox regression model comparing the OS of older and young patients, and adjusting for factors including having family history, nodal metastasis, M stage, LVI and grade III tumors, showed that older patients indeed had reduced survival. Patients of that cohort had an OS rate that was 1.6-times less than that of the younger patients (*p* = 0.0061, 95%CI = 1.145–2.252), with all the aforementioned factors showing significant interactions with the difference in OS between the elderly and the young (Table 2).

**Table 2:** Effect of clinical and pathologic variables on overall survival of older versus young patients.

Clinical/pathologic features		Hazard ratio	95% hazard ratio confidence limits		*p* value
Cohort	Older patients versus young patients	1.605	1.145	2.252	.0061
M stage	MI verus M0	5.596	3.409	9.187	< .001
Nodal metastasis	Positive versus negative	2.097	1.351	3.255	.0010
LVI	Positive versus negative	1.451	1.033	2.037	.0316
Triple negative	Yes versus no	1.899	1.132	3.185	.0151
Grade	III versus (I-II)	1.565	1.130	2.168	.0071

M = Metastasis; LVI = lymphovascular invasion.

## Discussion

As a developing country, Jordan is composed of a relatively young population, with 77.4% of its citizens being under the age of 40, and only 3.7% over the age of 65, according to the latest report by the Jordanian Department of Statistics^14^. The demographical distribution of the country’s citizens may therefore account for the observed younger median age at breast cancer diagnosis^15^. The local cancer registry reports reveal that 20.5% of all BC cases in Jordan occur in women younger than 40^16^, in contrast with the reported 10.2% of BC cases occurring before the age of 45 in the United States^10^. These observations, along with the relatively high rate of *BRCA1-* and *BRCA2-*associated BC cases reported among Jordanian patients, can be considered as additional key factors that contribute to a genetically-enriched landscape for BC disease patterns, with potential for distinctive age-defined outcomes^17,18^. This study managed to identify the ways by which the Jordanian BC population both adhered to, or deviated from, the global trends of age-related differences in breast cancer presentation and treatment outcomes.

In regards to the clinical presentation of young BC patients, the results of our study were, for the most part, similar to previously published data^19–22^, in that the younger patient group presented with several poor clinical indicators, including a stronger association with high-grade tumors, lymphovascular invasion and lymph-node involvement (Fig. 1). Moreover, we found that, when compared to the older patient cohort, younger patients were not only more likely to be HER2-positive, but also more likely to present with triple-positive disease (Table 1). Triple positive breast tumors constitute an emerging class of BC, that is increasingly demonstrating distinctive resistance patterns to both anti-HER2 targeting drugs and other endocrine therapies^23,24^. To our knowledge, this is the first study to portray a difference in the rate of the triple-positive tumor subtype between young and old BC patients. Triple-negative disease, on the other hand, was not significantly associated with the younger cohort. This is noteworthy because it deviates from previous data which has shown that patients of a younger age are at a higher risk for developing the more aggressive triple-negative disease^25–27^, indicating a possible unique trend in our population, and a potential avenue for investigation in our region.

Despite the fact that the aforementioned findings do present the younger patients as having clinical indicators of worse prognoses, the OS of the older cohort was significantly worse than that of the young (Fig. 3a). In fact, the older patients of our population also presented with unfavorable clinical properties. For instance, patients of the older group were more commonly diagnosed with distal metastasis and were more likely to have ILC, a type of breast tumor that has been associated with older patients and worse survival rates in the long-term^28^. A study by Li et al. similarly found that lobular carcinomas increased with age, and correlated ILC diagnosis with ER/PR + tumors^29^, which we also found to be more strongly associated with our older cohort (Table 1).

Despite a poor OS rate in older patient group, our data clearly showed that DFS is similar in both age groups (Fig. 3b), suggesting that the worse OS of the older patients might have actually resulted from the increased comorbidities and treatment differences that older populations globally suffer from. This conclusion is further supported by the fact that, even when excluding M1 patients, the 5-year OS rate of the older patients remained significantly worse than that of the young (Fig. 4). Although some studies reported reduced DFS and/or OS in their younger cohorts^7,30^, multiple have indeed found that despite the worse prognostic factors of their young BC cohorts, OS was still worse for their older patient groups^31–33^, and DFS was nondifferent between their cohorts as well^34^, challenging the utility of age as an independent prognostic risk factor.

The previous study that was conducted at our institution on the same group of older patients discussed the patients’ reduced rate of treatment by chemotherapy; specifically, that 32.8% of early-stage patients and 86.0% of those with metastatic disease never had chemotherapy^12^. In this study we revealed additional possible biases in terms of surgical intervention given to those patients, as the older patients received less surgery than the young cohort. This trend towards favoring less invasive approach to treatment of patients of an older age is globally reported^35,36^, and has proven to be non-standardized in that it tends to be based on patient and healthcare provider (HCP) preferences and fears of post-surgical complications^37^. This has important implications, especially when considering the body of evidence that establishes that surgical intervention is superior to hormonal therapy for older women with operable disease^38^. Moreover, a study by de Glas et al. has challenged this practice by proving that the lower survival rates of their older BC patients post-surgery were not caused by surgical complications, but were rather due to comorbidities or presence of concomitant diseases^39^. Additionally, the vastly reduced rate at which the older cohort underwent breast reconstruction surgeries can be a reflection of a less stringent psychological burden regarding body image on that age group of patients, this is also implied by their reduced rate of SSMs. The high frequency at which our older cohort underwent MRM and not BCS, when compared to the younger patients (Fig. 2), is likely to be also a reflection of favoritism by HCP for the use of MRM to treat older BC patients as an alternative to BCS with radiotherapy, which can be inconvenient or harmful to patients who suffer from co-morbid conditions^40,41^.

It is worth noting that the results of this study are both limited and aided by the nature of its design. Where, in basing it on previously collected data of adult BC patients ≤ 40 years old and that of BC cases of patients ≥ 65 years of age, we didn't include the BC cases of patients between the ages of 41 and 64. That being said, by restricting the comparisons in this study to patients who are truly older versus those who are young^42^, we were able to identify unique patterns of the disease, the opportunity of which may not have presented if we were limited by comparison at a single cut-off point of older or younger than 40 years, for example.

Ultimately, the findings of this study offer important implications on a number of aspects related to BC properties and their relationship with BC patient care in Jordan. Firstly, the atypical clinical characteristics of Jordanian young and older BC patients presented in our discussion are an added demonstration of the heterogeneity of the disease, further investigation is needed to determine whether these variances are indeed regional. Moreover, in spite of the controversy of the use of age as an independent prognostic marker for treatment outcomes and survival, it is apparent that in practice age is still a determinant factor in the choice of treatment.

Obviously, our study is not without limitations; the retrospective nature of the study and data collections from a single institution can be limiting factors. Additionally, there have been recent advances in the treatment of breast cancer in endocrine, targeted- and immuno-therapy for advanced-stage and triple negative disease, that might not be reflected in our cohort. Hopefully, the information we presented provide an incentive to HCPs to advocate more similar treatment protocols for young and old patients, particularly when it comes to surgical intervention for elderly BC patients who are otherwise healthy.

## Data Availability

The datasets generated during and/or analysed during the current study are available from the corresponding author on reasonable request.

## Abbreviations

BC: Breast cancer
DFS: Disease-free survival
ER: Estrogen receptor
HCP: Healthcare provider
HER2: Human epidermal growth factor receptor 2
ILC: Invasive lobular carcinoma
IRB: Institutional review board
M0: Cases without evidence of distal metastases
M1: Cases with distal metastases
MRM: Modified radical mastectomy
OS: Overall survival
PR: Progesterone receptor
SEER: Surveillance, epidemiology, and end results medicare database
SSM: Skin-sparing mastectomy
